# Low-Dose Interleukin-2 Enhances Activated Suppressor Regulatory T Cells and CTLA-4 and HLA-DR Expression in Chronic Chikungunya Arthritis

**DOI:** 10.3390/pathogens15070770

**Published:** 2026-07-22

**Authors:** Sarah R. Tritsch, Jose Forero Mejia, Evelyn Mendoza-Torres, Alfonso Sucerquia, Abebawork Adem, Juan David Alzate-Alvarez, Rimjhim Agarwal, Daniela Weiskopf, Edna Acosta, Estefanie Osorio-Llanes, Andres Orozco González, Alberto Panza Pallares, Marianna Carrillo Encinales, Victor Cañas Paez, Maria Jose Viera Contreras, Maria Jose Sarmiento Alvarez, Nicolle Suarez Otero, Diego Garcia Bañol, Lin Tan Kuang, Lucia Suárez Maestre, Belkis Meneses Rueda, Camilo Badel, Gary L. Simon, Gary S. Firestein, Liliana Encinales, Christopher Mores, Andres Cadena, Aileen Y. Chang

**Affiliations:** 1 Department of Global Health, Milken Institute School of Public Health, George Washington University, Washington, DC 20052, USA; sarahtritsch@email.gwu.edu (S.R.T.); cmores@email.gwu.edu (C.M.); 2 Department of Medicine, George Washington University, Washington, DC 20052, USA; jose.forero@email.gwu.edu (J.F.M.); alfonso.sucerquiahernandez@gwu.edu (A.S.); abebawork.adem@gwu.edu (A.A.); juan.alzate@gwu.edu (J.D.A.-A.); glsimonmd@outlook.com (G.L.S.); 3 Advanced Biomedicine Research Group, Faculty of Health, Exact and Natural Sciences, Universidad Libre de Colombia, Seccional Barranquilla, Barranquilla 080020, Atlántico, Colombia; evelyn.mendozat@unilibre.edu.co; 4 Center for Vaccine Innovation, La Jolla Institute for Immunology (LJI), La Jolla, CA 92037, USA; ragarwal@lji.org (R.A.); dweiskopf@lji.org (D.W.); 5 Biomedical Sciences Graduate Program, School of Medicine, University of California San Diego (UCSD), La Jolla, CA 92037, USA; 6 Division of Infectious Diseases and Global Public Health, Department of Medicine, University of California San Diego (UCSD), La Jolla, CA 92037, USA; 7 Allied Research Society, Barranquilla 080020, Atlántico, Colombia; ednaacosta2009@gmail.com (E.A.); aorozco@alliedresearchcol.com (A.O.G.); apanza@alliedresearchcol.com (A.P.P.); marianna.carrillo2004@gmail.com (M.C.E.); vcanas@alliedresearchcol.com (V.C.P.); nicolleso59@gmail.com (N.S.O.); camilo.badel@umm.de (C.B.); liliana_encinales@yahoo.com (L.E.); 8 Centro de Investigación, Clínica de la Costa SAS, Barranquilla 080020, Atlántico, Colombia; mariajoseviera27@gmail.com (M.J.V.C.); msarmientoj23@gmail.com (M.J.S.A.); dr.diegogarciab@gmail.com (D.G.B.); kuanglin123@gmail.com (L.T.K.); lucia.suarezmaestre@gmail.com (L.S.M.); belkisrm100@gmail.com (B.M.R.); acadena@clinicadelacosta.co (A.C.); 9 Department of Medicine, UC San Diego School of Medicine, San Diego, CA 92093, USA; gfirestein@health.ucsd.edu; 10 Department of Medicine, Universidad Simón Bolívar, Barranquilla 080020, Atlántico, Colombia

**Keywords:** chikungunya, arthritis, regulatory T cells, CTLA-4, interleukin-2

## Abstract

Chronic chikungunya arthritis is a debilitating post-viral inflammatory arthritis with no established evidence-based therapy. Regulatory T cell (Treg) dysfunction may contribute to persistent inflammation, and low-dose interleukin-2 (IL-2) may restore immune regulation. We evaluated the immunomodulatory effects of low-dose IL-2 using peripheral blood mononuclear cells from adults with laboratory-confirmed chronic chikungunya arthritis in Atlántico, Colombia. Cells were treated ex vivo with recombinant IL-2 or an IL-2/anti-IL-2 monoclonal antibody complex in the presence of CD2/CD3/CD28 stimulation beads, followed by flow cytometric assessment of effector T cells and Tregs. Low-dose IL-2 treatments ex vivo did not increase total Treg frequency but selectively increased activated suppressor Tregs while decreasing activated T effector cells, enhancing Treg CTLA-4 and HLA-DR expression, and reducing Ki67 expression in effector T cells. IL-2 complex treatment decreased cytokine-secreting Tregs, and IL-10 and TGF-β were not associated with IL-2 treatment status or with Teff/Treg balance, suggesting limited utility as pharmacodynamic biomarkers of low-dose IL-2 treatments. These findings support the use of activated suppressor Tregs, Treg CTLA-4, and HLA-DR expression as candidate biologic endpoints for evaluation in future trials of low-dose IL-2 in chikungunya arthritis.

## 1. Introduction

Chikungunya virus (CHIKV) was introduced into the Western Hemisphere for the first time in 2013 and has caused over three million infections across more than 119 countries globally since then, including local outbreaks reported in the United States [1]. Approximately one fourth of infected individuals developed chronic, debilitating arthritis [2]. CHIKV is one of several arthritogenic alphaviruses, a group that also includes Mayaro, O’nyong-nyong, Ross River, and Sindbis viruses [3]. Despite the substantial burden of the disease, there is no standard, evidence-based treatment for chronic CHIKV arthritis or for alphaviral arthritis more broadly.

For chronic chikungunya arthritis that does not respond to short courses of nonsteroidal anti-inflammatory drugs (NSAIDs) and corticosteroids, methotrexate is sometimes used [4]. A placebo-controlled trial of methotrexate is underway (NCT04483466); however, methotrexate is not an ideal solution. Methotrexate is teratogenic [5], and chikungunya arthritis disproportionately affects women, with approximately half of affected women in their childbearing years [2]. In addition, hepatic and hematologic toxicities may limit its use [6,7]. These limitations highlight the need for safer and more targeted therapies.

Emerging data suggest that targeting regulatory T cells (Tregs) with low-dose interleukin (IL)-2 may represent a promising therapeutic strategy for chronic chikungunya arthritis. Preliminary studies indicate that altered Treg function may contribute to CHIKV arthritis pathogenesis. Tregs regulate immune responses by suppressing effector CD4+ T cells (Teff), and inadequate Treg activity may predispose individuals to arthritis by permitting dysregulated inflammation. CHIKV arthritis is associated with infiltration of large numbers of Teff cells into the synovium [8,9], and in mouse models, Teff cells are required for the development of arthritis [8]. In parallel, CHIKV infection has been associated with reduced Treg frequency [10,11,12] and impaired expression or production of key Treg functional markers, including cytotoxic T lymphocyte antigen (CTLA)-4, CD39-CD73, perforin, granzyme, programmed death 1 (PD-1), and transforming growth factor (TGF)-β, during both the acute and chronic phases of the disease [11].

Although high-dose IL-2 therapy is associated with substantial toxicity, low-dose IL-2 preferentially activates Tregs because the IL-2 receptor on Tregs has an approximately 20-fold lower activation threshold than the IL-2 receptor on Teff cells, thereby promoting selective Treg expansion and activation [13]. In rheumatoid arthritis, low-dose IL-2 has been shown to increase Treg frequencies, reduce disease activity, and produce no apparent major side effects [14]. These findings suggest that low-dose IL-2 may also have therapeutic potential in CHIKV arthritis.

Murine studies further support this approach. In mice with CHIKV arthritis, treatment with low-dose IL-2 complexed to the monoclonal antibody JES6-1 increased activated Treg levels [15]. This antibody reduces IL-2 degradation and promotes preferential binding of IL-2 to the high-affinity IL-2 receptor expressed on Tregs. Furthermore, JES6-1 alone increased activated Treg levels, enhanced Forkhead box P3 (FOXP3) expression, a key mediator of Treg suppressive function, and improved histologic markers of inflammation, possibly through augmentation of endogenous IL-2 activity [15].

However, the mechanisms underlying IL-2-based therapies in human CHIKV arthritis remain undefined, and this knowledge gap limits rational phase II clinical trial development. Our study focuses on immune regulation, specifically whether IL-2-based strategies can expand and activate Tregs and rebalance inflammation in post-CHIKV chronic arthritis. The goal of this study was to define the mechanisms of low-dose IL-2-induced immunosuppression in human chronic chikungunya arthritis and to identify measurable biologic endpoints to inform a phase II clinical trial of low-dose IL-2 therapy in this population.

## 2. Materials and Methods

### 2.1. Participants and Setting

Participants were recruited from Atlántico, Colombia, where a local CHIKV epidemic began in 2014. Participants aged 18 and older with laboratory-confirmed CHIKV infection were analyzed. Participants were recruited from 12 March to 26 July 2024.

### 2.2. Study Design

Participants with a clinical diagnosis of chikungunya and a history of CHIKV infection confirmed by ELISA IgM, IgG, or PCR were screened. As per the Colombian Institute of Health, a clinically confirmed case of CHIKV infection is defined as a fever of >38 °C, severe joint pain or arthritis, and the acute onset of erythema multiforme with symptoms not explained by other medical conditions. In addition, these individuals must reside in or have visited a municipality where evidence of CHIKV transmission is present or have traveled within 30 km of confirmed viral circulation. Diagnosis of CHIKV was serologically confirmed via IgG antibody immunofluorescence, as described below. Participants were excluded if CHIKV IgG was negative. A face-to-face history and physical examination were performed to collect demographic data, exposure history, and signs and symptoms of arthritis. Participants were included in the chronic arthritis group if they self-reported symptoms of articular pain and demonstrated evidence of clinical synovitis for at least three months. A clinical history was collected to further understand the temporal relationship between CHIKV infection and arthritis initiation and the prevalence of comorbid arthritis prior to CHIKV infection. Blood samples were collected for cytokine analysis and in vitro experimentation.

### 2.3. Arthritis Disease Activity, Disability and Patient-Reported Outcome Measures

Arthritis disease activity was assessed using the Disease Activity Score in 28 joints (DAS-28) [16] with C-reactive protein (CRP), which incorporates the number of tender and swollen joints (of the 28 assessed), CRP level, and a patient global health assessment on a visual analog scale. DAS-28 scores were categorized as remission (<2.6), low disease activity (2.6–3.2), moderate disease activity (3.2–5.1), or high disease activity (>5.1).

Arthritis flare activity was assessed using an adapted version of the Outcome Measures in Rheumatology Rheumatoid Arthritis Flare Questionnaire (OMERACT-FQ) [17] for patients with CHIKV arthritis. The instrument included five items evaluating pain, physical function, stiffness, fatigue, and participation over the prior week, rated on 11-point numeric rating scales (0 = none, 10 = severe). The composite score, calculated as the sum of the five domain scores, ranged from 0 (no flare) to 50 (extreme flare). Although flares are generally defined by a total score > 25, symptoms lasting more than 1 week, and patient self-classification of a flare, this study analyzed the score as a continuous variable, as recommended.

Disability was assessed using the Health Assessment Questionnaire Disability Index (HAQ-DI) [18], a measure of physical function across 20 items in 8 domains: dressing, rising, eating, walking, hygiene, reach, grip, and usual activities. Each item is scored on a 4-point scale from 0 to 3, where 0 indicates no difficulty, 1 some difficulty, 2 much difficulty, and 3 inability to perform the activity. HAQ-DI scores are categorized as mild to moderate disability (0–1), moderate to severe disability (1–2), and severe to very severe disability (2–3).

Finally, the Patient-Reported Outcomes Measurement Information System (PROMIS) measures were used to assess quality of life [19]. PROMIS-29 was used to assess seven domains (physical function, anxiety, depression, fatigue, sleep disturbance, satisfaction participating in social roles and pain interference) with four questions for each domain evaluated over the previous seven days.

### 2.4. Data Management

Each participant was assigned a unique study identification number used for database records and sample labeling. All data were de-identified and stored in the REDCap database at The George Washington University.

### 2.5. Sample Collection and Processing

Blood was collected via venipuncture into sodium heparin vacutainers (BD Biosciences, San Jose, CA, USA). The blood samples were centrifuged at room temperature (18–25 °C) in a horizontal rotor for 20 min at 1500 relative centrifugal force. Plasma was stored at −80 °C until analysis. Peripheral blood mononuclear cells (PBMCs) were separated using Lymphoprep and SepMate tubes from STEMCELL Technologies (Cambridge, MA, USA) and cryopreserved.

### 2.6. Chikungunya IgG Immunofluorescence

An immunofluorescence assay (Euroimmun, Lübeck, Germany) was used to detect anti-chikungunya virus IgG antibodies in enrolled patients. Plasma samples were applied to slides containing biochips with chikungunya-positive and chikungunya-negative cells. When present, anti-CHIKV IgG bound to the positive cells and produced fluorescence. Slides were examined on a Biotek Lionheart LX fluorescent microscope (Agilent, Santa Clara, CA, USA) using a 488 nm excitation laser and a 4× objective. The assay has a reported sensitivity of 97% and specificity of 96%.

### 2.7. IL-2 Treatment

PBMCs were isolated from patients as described above and frozen until analysis. After thawing, PBMCs were washed once with DMEM containing FBS and then rested overnight. CD4+ cells were sorted using the MojoSort Human CD4 T cell Isolation Kit (Biolegend, San Diego, CA, USA). The CD4+ cell fraction was counted in AOPI staining solution (Nexcelom, Lawrence, MA, USA), distributed evenly into 24-well plates, and then incubated with Treg Suppression Inspector beads containing biotinylated CD2, CD3, and CD28 antibodies (Miltenyi Biotec, Gaithersburg, MD, USA) at a ratio of one bead to one cell, as well as 25 IU/mL of recombinant IL-2 or a complex containing IL-2 and an anti-IL-2 human monoclonal antibody (BD Biosciences, San Jose, CA, USA) that prevents degradation of IL-2 and causes preferential binding to the high-affinity IL-2 receptor on Tregs [20]. The purpose of Treg Suppression Inspector beads is to serve as an optimized stimulation reagent for in vitro assays to activate responder T cells while enabling the functional characterization of human Tregs. IL-2 treatments were refreshed after 48 h, and cells were collected 96 h after the first treatment for flow cytometry analysis. Cell supernatants were collected for cytokine analysis.

### 2.8. Cytokine Analysis

Cytokines, including IL-10 and TGF-β, were measured using the V-PLEX IL-10 Assay and U-PLEX Human TGF-β1 Assay Kits from Meso Scale Diagnostics (Rockville, MD, USA) according to the manufacturer’s instructions on patient plasma and the in vitro supernatant.

### 2.9. Flow Cytometry

Flow cytometry was performed on a Cytek Aurora (Fremont, CA, USA) flow cytometer and analyzed in FlowJo 11 (TreeStar Inc., Ashland, OR, USA). T cells were stained using antibodies against the extracellular markers CD4, CD25, CD38, CCR7, CD73, PD-1, CTLA-4, CD45RO, CD45RA, CD122, CD39, and HLA-DR, as well as the intracellular markers granzyme A, perforin, FOXP3, and Ki67 (Biolegend, San Diego, CA, USA). Live/Dead Fixable Aqua Dead Cell Stain and FOXP3 Fixation/Permeabilization Buffer were obtained from ThermoFisher Scientific (Waltham, MA, USA).

All antibodies were titrated before use. Single antibody-stained PBMCs were used for compensation, and fluorescence minus one (FMO) controls were performed to assist with gating. Duplicate and dead cells were removed from the analysis using single-cell and live-cell gates. Tregs were defined as CD3+CD4+CD25hi/int+FOXP3+, and Teff were defined as CD3+CD4+CD25low/−.

### 2.10. Statistical Analysis

R (version 4.4.1) and GraphPad Prism (version 11) were used to analyze and graph data. A *p*-value of <0.05 was considered statistically significant. Analyses comparing treatment groups were performed using a repeated-measures analysis of variance (ANOVA), followed by Dunnett’s post-test comparing each treatment group to the control (beads only) group. A Welch’s *t*-test was used to analyze differences between specific groups, such as NSAID use. To evaluate relationships between cytokine concentrations and clinical and immunological parameters, treatment-stratified correlation analyses were performed using Spearman’s rank correlation coefficients. Correlations were assessed separately within each treatment condition (control, IL-2, and complex) to account for treatment-specific effects. Variables examined included IL-10 and TGF-β1 concentrations in relation to the Treg:Teff ratio, regulatory T cell frequency, effector T cells and CTLA-4-expressing regulatory T cells; Correlation coefficients (ρ) and corresponding *p*-values were reported. For comparison of Treg and cytokine levels, repeat measures ANOVA with a moderate effect size (f = 0.256) and alpha= 0.05 with 50 cases per group provided a power of 0.80. Therefore, the study was powered to detect moderate-to-large effects but not smaller effects.

## 3. Results

The majority of the study population was female (62%) with a median age of 45.0 (IQR 31.2–56.5) (Table 1). Median disease activity was low as per the Disease Activity Score-28. Most of the population was not in a current arthritis flare at the time of sampling, indicated by a modified OMERACT flare score for chikungunya arthritis. Median disability scores indicated that most participants had some difficulty as measured by the health assessment questionnaire. Prior arthritis before chikungunya infection was uncommon, reported by 2/50 participants (4.0%), and the median time from reported chikungunya infection to arthritis onset was 12 days (IQR 4–32). The most frequently reported therapies were acetaminophen/paracetamol (39/50, 78.0%) and NSAIDs (22/50, 44.0%). Corticosteroid/prednisone use was uncommon (4/49, 8.2%), and methotrexate use was rare (1/49, 2.0%). Approximately one quarter (13/49, 26.5%) reported alternative medicine use that included reported use of cane sugar, lime, and eucalyptus.

### 3.1. IL-2 and Complex Treatments Significantly Decrease Teff Cell Proliferation

A concentration of 25 IU/mL was chosen based on the literature and a preliminary dose–response study using healthy donor PBMCs (Supplementary Figure S1). Lee et al. (2016) reported the response of both Tregs and Teff populations when healthy donor PBMCs were treated with concentrations of 1–1000 ng/mL IL-2 [21]. In addition, a study in type-1 diabetes patients found that treatment with 1–1.5 × 10^6^ units/m^3^ IL-2 produced a maximum concentration of 25 IU/mL IL-2 in peripheral blood at 90 min post-infusion [22]. Our preliminary ex vivo study tested IL-2 at 12.5, 25, and 50 IU/mL. All concentrations increased the percentage of Tregs and activated Tregs (CD45RA-HLA-DR+) out of CD4+ cells and the percentage of Ki67+ Tregs compared to untreated cells, with the highest response occurring at 25 IU/mL. Therefore, we chose the middle concentration of 25 IU/mL (approximately 1.5 ng/mL) IL-2.

**Figure 1:**
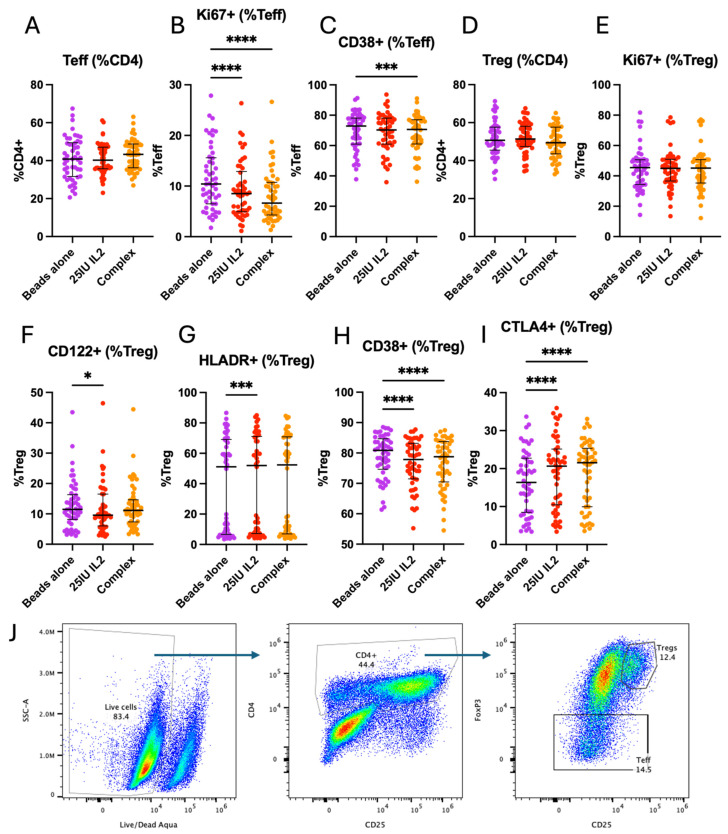
The effect of IL-2 treatment on Teff and Treg cell frequency, proliferation, and activity. Flow cytometry was performed on IL-2 or complex-treated CD4+ cells to analyze the effect of treatment on Teff and Treg cell frequency and proliferation. Control wells were incubated with Treg Suppression Inspector beads alone. Lines on the graphs denote the median with IQR. The percent of Teff (**A**) and Treg (**D**) cells out of the CD4+ cells did not change with treatments. Cell proliferation determined by percent Ki67+ decreased with treatment in Teff cells (**B**), but not in Treg cells (**E**). CD38, an activation marker, decreased with complex treatment on Teff (**C**) and with both treatments on Tregs (**H**). CD122 (**F**) and HLADR (**G**) were significantly different on Tregs with IL2 treatment only, and CTLA4 (**I**) increased on Tregs in both treatment groups. The gating strategies for Tregs and Teffs using CD4, CD25, and FOXP3 are displayed (**J**). Arrows indicate gates used to form subsequent plots. * *p* < 0.05, *** *p* < 0.001, **** *p* < 0.0001.

PBMCs from post-CHIKV arthritis patients were treated with either IL-2 or complex in the presence of Treg Suppression Inspector Beads. The percentage of CD4+ T effector cells (Teffs) and T regulatory cells (Tregs) as a proportion of live PBMCs and the frequency of Ki67, a proliferation marker, were analyzed, as well as markers that suggest cell activity. The average percent of Teff and Treg cells out of CD4+ cells did not change with IL-2 or complex treatments compared to beads alone (Figure 1A,D). Proliferation of Tregs also did not change, since the percent of Ki67+ Tregs remained the same (Figure 1E). However, the Ki67+ proliferation of Teff cells decreased significantly with both IL-2 and complex treatments, compared to beads alone (*p* < 0.0001, Figure 1B).

### 3.2. Significant Increase in CD38+ and CTLA-4+ Tregs with IL-2 and Complex Treatments

Treg activation markers were examined via flow cytometry after IL-2 or complex treatments. Many remained unchanged in the presence of IL-2 or complex treatments, including CCR7, CD39/CD73, perforin, granzyme, and PD-1 (Supplementary Figure S2). There was lower expression of the IL-2 receptor CD122 (*p* = 0.014) that is part of both the lower-affinity dimeric IL-2 receptor found on conventional T cells and the high-affinity trimeric receptor found primarily on Treg cells and the activation marker HLA-DR (*p* = 0.014) in Tregs in the presence of IL-2 compared to beads alone (Figure 1F,G). CD38 and CTLA-4 were the only activation markers that increased in Tregs with IL-2 and complex treatments compared with beads alone (*p* < 0.0001 for all, Figure 1H,I).

**Figure 2:**
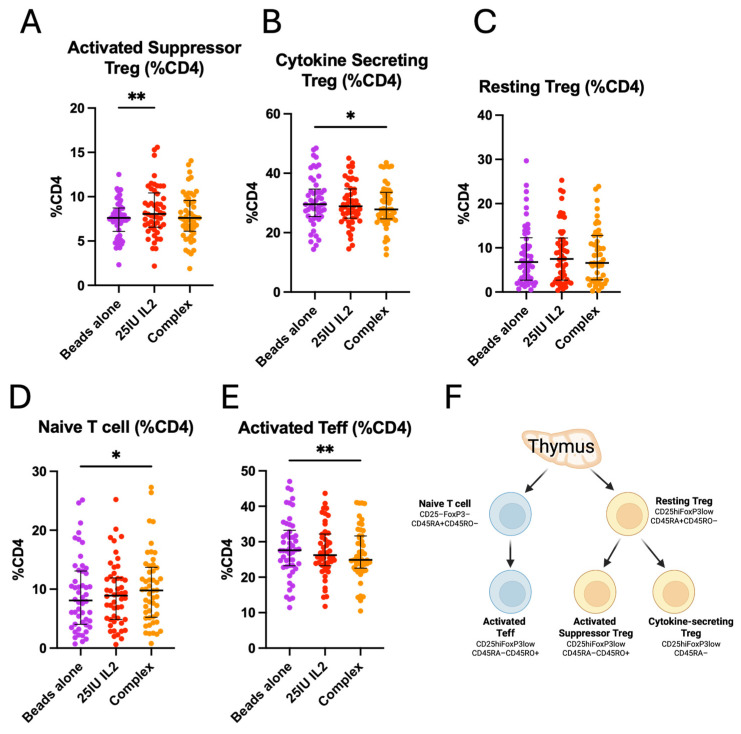
The effect of treatment on Treg cell activity. Flow cytometry was performed on IL-2- or complex-treated CD4+ cells to analyze the effect of treatment on Treg cell activity. Control wells were incubated with Treg Suppression Inspector beads alone. Lines on the graphs denote the median with IQR. Activated suppressor Tregs (**A**) increased with IL2 compared to beads alone; cytokine-secreting Tregs (**B**) and activated Teffs (**E**) decreased with complex treatment; and naïve T cells (**D**) increased with complex treatment. Resting Tregs (**C**) did not change with treatment. (**F**) Schematic depicting how T cell (blue) and Treg (yellow) subpopulations were classified using CD25, FOXP3, CD45RA, and CD45RO. * *p* < 0.05, ** *p* < 0.01.

Additionally, CD4+ T cells were subdivided into resting Tregs (CD25hiFOXP3lowCD45RA+CD45RO−), activated suppressor Tregs (CD25hiFOXP3hiCD45RA−CD45RO+), cytokine-secreting Tregs (CD25hiFOXP3lowCD45RA−), naïve T cells (CD25−FOXP3−CD45RA+CD45RO−), and activated Teff cells (CD25hiFOXP3lowCD45RA−CD45RO+) (Figure 2). There were no significant changes in resting Tregs, while activated suppressor Tregs increased slightly but significantly compared to the control when cells were treated with IL-2 (*p* = 0.0012). Conversely, naïve T cells increased (*p* = 0.03) with complex treatment, while cytokine-secreting Tregs (*p* = 0.013) and activated Teff cells (*p* = 0.01) decreased.

Subgroup analysis was performed to determine the effect of disease status and NSAID or acetaminophen/paracetamol use on important cell populations. Reported NSAID or acetaminophen/paracetamol use did not significantly change the frequency of Tregs, CTLA4+ Tregs, Ki67+ Teff, or activated suppressor Treg populations. The results were categorized by disease activity score: remission (DAS28 < 2.6, *n* = 20), low (DAS28 = 2.6–3.2, *n* = 12), or moderate to severe (DAS28 > 3.2, *n* = 17). None of the categories showed significantly different responses to treatment across most of the cell populations. When examining only patients with moderate-to-high disease scores, IL2 treatment significantly increased the percentage of CTLA4+ Tregs (*p* < 0.0001) compared with beads alone, whereas the complex treatment significantly decreased the percentage of Ki67+ Teff cells (*p* = 0.008, Supplementary Figure S3).

### 3.3. Cell Supernatant Analysis of IL-10 or TGF-β Does Not Correlate with Markers of Treg Immunosuppression in CHIKV Arthritis

**Figure 3:**
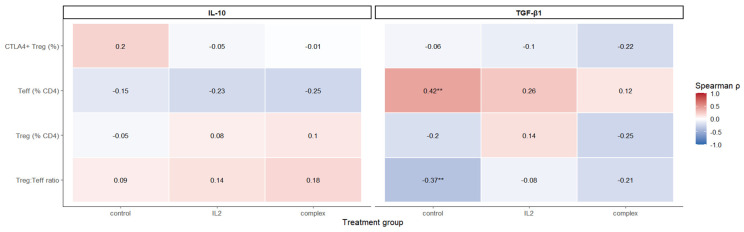
Treatment-stratified correlations between cytokines and immune parameters. Correlations between cytokines (IL-10 and TGB-β) and immune parameters, including Treg/Teff ratio, percent Treg of CD4+ T cells, percent Teff of CD4+ T cells, and percent CTLA-4+ Treg, are shown for control (Treg Suppression Inspector beads alone), IL-2- and complex-treated groups in a heat map format. ** *p* < 0.01.

Treatment-stratified Spearman correlation analyses were conducted to examine associations between cytokine concentrations (IL-10 and TGF-β1) and immunological parameters, including the Treg (%), Teff (% CD4), Treg (% CD4), Treg:Teff ratio, and CTLA-4 expression across treatment groups, to examine the potential relationship of IL-2 treatments to cytokine levels (Figure 3). Across all treatment groups, IL-10 concentrations did not demonstrate statistically significant correlations with any of the examined immunological parameters, including Treg frequency, Teff frequency, Treg:Teff ratio, or CTLA-4+ Tregs. In contrast, TGF-β1 concentrations showed associations with effector T cell measures, but only in the control group. In the control condition, TGF-β1 was positively correlated with Teff (% CD4+) (ρ = 0.42, *p* = 0.002) and negatively correlated with the Treg:Teff ratio (ρ = −0.37, *p* = 0.008).

## 4. Discussion

The goal of this study was to define the mechanisms of low-dose IL-2-induced immunosuppression in chronic chikungunya arthritis (CHIKA) using human samples and to identify measurable candidate biologic endpoints for a future phase II clinical trial. Our primary finding was that low-dose IL-2 did not increase the overall Treg population (CD3+CD4+CD25hi/int+FOXP3+). Instead, IL-2 selectively increased activated suppressor Tregs (CD25hiFOXP3hiCD45RA−CD45RO+) and was associated with decreased expression of proliferation marker Ki67 on effector T cells (Teff). To our knowledge, this is the first study to specifically evaluate CD25hiFOXP3hiCD45RA−CD45RO+ activated suppressor Tregs during low-dose IL-2 therapy. This subset increased with IL-2 but not with the IL-2/antibody complex. The mechanism is unclear, but the complex may preferentially expand less differentiated Tregs or alter IL-2 receptor engagement, signaling kinetics, or tissue trafficking, thereby limiting expansion of this circulating activated Treg population. These findings suggest that the mechanistic immunomodulatory effects of low-dose IL-2 in CHIKA may not be mediated by expansion of total Tregs, but perhaps by enrichment of a functionally suppressive activated Treg subset, likely through a possible CTLA-4- or HLA-DR-dependent mechanism. CTLA-4 is a co-inhibitory receptor in the CD28 family, is constitutively expressed on Tregs, and is a key pathway by which they suppress immune activation. In this study, we demonstrated that CTLA-4 expression was augmented through IL-2 therapies ex vivo. Performing a functional suppression assay or using a CTLA-4 blocking antibody was not possible with this cohort, but this should be used in future studies to further investigate this mechanism. Additionally, HLA-DR is expressed by approximately one third of effector Tregs in adult humans. HLA-DR+ Tregs express higher levels of FOXP3 and are responsible for contact-dependent in vitro suppression, while the suppression by HLA-DR−Tregs is initially mediated by IL-4 and IL-10 and later by a contact-dependent mechanism. HLA-DR+Treg cells are terminally differentiated and highly suppressive Tregs [23]. Therefore, CTLA-4 and HLA-DR mechanisms are plausible mechanisms for low-dose IL-2-mediated Treg suppression.

Our findings further help us to define a biologically relevant dose range for future investigation. In our study, 25 IU/mL IL-2 (which is approximately 1.5 ng/mL) was sufficient to activate suppressor Tregs ex vivo from peripheral blood mononuclear cells from patients with chikungunya arthritis. This is consistent with prior studies showing that human memory T cells (CD45RA-) from healthy donors respond to IL-2 concentrations between 1 and 100 ng/mL and that 50 IU/mL IL-2 expands functionally suppressive CD4+ regulatory T cells in vitro through a STAT5-dependent pathway in patients with chronic kidney disease [24]. Together, these data suggest that relatively low concentrations of IL-2 can induce meaningful immunoregulatory effects across disease settings. Given that low-dose IL-2 is a relatively safe and targeted therapy [13,14], these findings support its further evaluation in phase II clinical trials for alphaviral arthritis to further define ideal dosing.

Many studies have described the proinflammatory immune environment of acute CHIKV infection, and data suggest that persistent cytokine dysregulation could be linked to chronic arthritis [25], including the development of an immune phenotype that maintains synovial inflammation. Utilizing the supernatants, we also evaluated transforming growth factor beta (TGF-β) and IL-10 to determine whether the release of these cytokines could serve as practical biomarkers of the immunomodulatory effects of low-dose IL-2 in clinical trials. We showed that cytokine-secreting Tregs (CD25hiFOXP3lowCD45RA−) were decreased by IL-2 complex treatment, and in vitro TGF-β and IL-10 levels did not correlate with the proportions of Teff, Treg, or the Treg/Teff ratio after IL-2 therapy. In addition, in our in vitro study, these cytokine levels were not associated with IL-2 treatment status, and CTLA-4 expression on Tregs did not correlate with either TGF-β or IL-10. Taken together, these results suggest that TGF-β and IL-10 are unlikely to be useful pharmacodynamic biomarkers of low-dose IL-2 activity in CHIKA clinical trials; however, additional larger cytokine profiling may be helpful. In a prior study with participants with chikungunya arthritis (*n* = 158) [26], IL-2 levels and frequency of Tregs were low. Increased arthritis disease activity was associated with higher levels of inflammatory cytokines (IL-6, TNF and CRP) and immunoregulatory cytokine IL-10 (*p* < 0.05). Further analysis of the role of the effect of low-dose IL-2 therapies on these cytokines is needed. Conforti et al. reported that patients who develop chronic chikungunya rheumatologic manifestations may exhibit an early proinflammatory profile characterized by elevations in IL-6, IL-8, TNF-α, IFN-γ, and interferon-inducible chemokines, with persistent IL-6, IL-8, TNF-α, and IL-17 signaling in some patients with chronic symptoms [25]. These findings complement our results by suggesting that inadequate regulatory control may fail to counterbalance an inflammatory program established early in infection, thereby providing further rationale for low-dose IL-2 therapy to restore Treg-mediated immune homeostasis in chronic chikungunya arthritis. Furthermore, we propose that activated suppressor Tregs (CD25hiFOXP3hiCD45RA−CD45RO+) and Treg CTLA-4 and HLA-DR expression are more appropriate possible candidate biomarkers for future studies. Validation of biomarkers in early-phase clinical trials is needed.

Subgroup analyses were performed to determine the effect of medication on Treg frequencies and markers. Reported NSAID use did not significantly change the Treg frequency, CTLA4+ Tregs, Ki67+ Teff, or the activated suppressor Treg populations. The dosages, frequencies and formulations of NSAIDs were not standardized, as this was not an interventional trial. Similarly, these outcomes did not appear altered by acetaminophen/paracetamol use; however, statistical significance was limited by only having 11 patients not reporting acetaminophen use. Corticosteroid use was only reported in four patients and methotrexate use in only one patient. Therefore, statistical comparisons could not be performed in those groups. Future clinical trials should consider the data collection on the use of concomitant medications.

When the results were categorized by disease activity score (remission, low, or moderate to severe), no one category showed significantly different responses to treatment across multiple cell populations. Clinically, only moderate-to-high disease scoring would be treated with pharmacological agents. Focusing on those groups, IL2 significantly increased the percentage of CTLA4+ Tregs compared to the control, and the complex significantly decreased the percentage of Ki67+ Teff cells compared to the control. Importantly, while DAS28 is commonly used to assess CHIKA disease, it was created to measure RA disease activity, and as such, it focuses on swelling of joints commonly affected by RA. Conversely, the smaller joints are more commonly affected in CHIKV, while stiffness without swelling is associated with disability and decreased quality of life. Therefore, DAS28 likely underestimates CHIKA disease activity. A CHIK-DAS specialized scoring system, such as the one we published in 2024, could more accurately capture this data [27].

This study has several limitations. First, T cell stimulation was performed using beads rather than antigen-presenting cells. Although this reductionist system does not fully replicate physiologic immune interactions, it still allowed us to demonstrate a CTLA-4-regulated suppressive effect. Second, we were unable to assess the longer-term effects of IL-2 exposure because cell viability declined with extended culture, particularly in cryopreserved samples. We were also unable to perform a direct functional suppression assay due to the limited volume of PBMCs available from this cohort. Future studies using fresh, non-cryopreserved cells may allow evaluation of more sustained IL-2-mediated effects and the ability to perform functional assays such as CTLA-4 blocking or Treg suppression assays. Due to the limited amount of samples, we report the change in T cells overall but have not measured CHIKV-specific T cells. Future studies should evaluate CHIKV-specific T cell responses in chikungunya arthritis cohorts. Finally, responses varied across participants, suggesting biologic heterogeneity in IL-2 responsiveness. Larger studies will therefore be needed to define responder and non-responder subgroups and to identify clinical or immunologic factors associated with treatment response.

## 5. Conclusions

Low-dose IL-2 selectively expanded activated suppressor Tregs and increased CTLA-4 and HLA-DR expression while reducing effector T cell proliferation marker Ki67 in ex vivo experiments, supporting the need for further clinical studies to evaluate the restoration of immune regulation as a potential therapeutic mechanism in chronic chikungunya arthritis. However, TGF-β and IL-10 did not correlate with IL-2-related changes in T cell subsets and cytokine-secreting Tregs were decreased by IL-2 complex therapy; therefore, these cytokines may be unlikely to serve as useful mechanistic pharmacodynamic biomarkers of IL-2 therapy. These findings identify activated suppressor Tregs and Treg expression of CTLA-4 and HLA-DR as the most relevant potential biologic endpoints for future studies and provide a mechanistic rationale for future phase II trials of low-dose IL-2 in chikungunya arthritis and potentially other alphaviral arthritides.

## Figures and Tables

**Table 1:** Demographic and clinical characteristics of the study population (*n* = 50).

Variable	Median (IQR) Except for Sex Variable
Age (years)	45 (31.2, 56.5)
Female, *n* (%)	31/50 (62.0%)
Disease activity by the DAS-28	2.8 (1.8, 3.8)
Arthritis flare activity by the modified OMERACT score for chikungunya arthritis	9.6 (0.4, 19.8)
Disability by the HAQ score	0.2 (0.0, 0.5)
Prior arthritis before chikungunya infection	2/50 (4.0%)
Time from reported chikungunya infection to arthritis onset (days) (*n* = 40)	12 (4.0, 32.0)
Arthritis duration (months) (*n* = 40)	108.2 (90.0, 115.5)
Medication Use	
NSAID use	22/50 (44.0%)
Acetaminophen/paracetamol use	39/50 (78.0%)
Corticosteroid/prednisone use	4/49 (8.2%)
Methotrexate use	1/49 (2.0%)
Alternative medicine use	13/49 (26.5%)

DAS, Disease activity score; HAQ, Health assessment questionnaire; OMERACT, Outcome measures in rheumatology.

## Data Availability

The data generated or analyzed during this study are included in this article and its Supplementary Materials files.

## Supplementary Materials

The following supporting information can be downloaded at https://www.mdpi.com/article/10.3390/pathogens15070770/s1, Figure S1: Determination of IL-2 concentration using healthy donor cells. Healthy donor PBMCs were treated with 12.5, 25, or 50 IU/mL IL-2, refreshed after 48 h, and collected and stained for flow cytometry after 96 h. Tregs (A), activated Tregs (B), Granzyme A+ Tregs (C), and Ki67+ Tregs (E) all increased with treatment compared to no treatment, with slightly greater changes in Tregs, activated Tregs, and Ki67 using 25 IU/mL. CTLA4+ Tregs (D) decreased with increasing amounts of IL2. Based on this data, 25 IU/mL was chosen for all subsequent experiments. Figure S2: No effect of treatment on certain markers of Treg cell activity. Flow cytometry was performed on IL-2 or complex-treated CD4+ cells to analyze the effect of treatment on Treg cell activity, including CCR7 (A), CD38/CD73 (B), perforin (C), granzyme A (D), and PD-1 (E). Control wells were incubated with Treg Suppression Inspector beads alone. Lines on the graphs denote the median with IQR. Comparisons between groups were all non-significant. Figure S3: Effect of treatments on PBMCs from patients with moderate-to-high disease score. When observing only patients with moderate-to-high disease scores (DAS28 > 3.2), comparisons show an increase in CTLA4+ Tregs (B) when PBMCs were treated with IL2 and a decrease in Ki67+ Teff (C) with complex treatment compared to Treg Suppression Inspector beads alone. Other important populations that were analyzed, including total Tregs (A), activated suppressor Tregs (D), activated Teffs (E), cytokine-secreting Tregs (F), and naïve T cells (G), showed no significant changes from the beads alone control. Lines on the graphs denote the median with IQR.

## Abbreviations

ANOVA	analysis of variance
CCR7	C-C chemokine receptor 7
CD	cluster of differentiation
CHIKA	chronic chikungunya arthritis
CHIKV	chikungunya virus
CRP	C-reactive protein
CTLA-4	cytotoxic T lymphocyte antigen 4
DAS-28	Disease Activity Score in 28 joints
DMEM	Dulbecco’s modified Eagle medium
FBS	fetal bovine serum
FOXP3	forkhead box P3
FWA	Federalwide Assurance
GWU	George Washington University
HAQ	Health Assessment Questionnaire
HAQ-DI	Health Assessment Questionnaire Disability Index
HLA-DR	human leukocyte antigen–DR
IgG	immunoglobulin G
IL-2	interleukin-2
IL-10	interleukin-10
IQR	interquartile range
IRB	Institutional Review Board
IU	international unit
NSAIDs	nonsteroidal anti-inflammatory drugs
OMERACT	Outcome Measures in Rheumatology
OMERACT-FQ	Outcome Measures in Rheumatology Rheumatoid Arthritis Flare Questionnaire
PBMCs	peripheral blood mononuclear cells
PD-1	programmed cell death protein 1
PROMIS	Patient-Reported Outcomes Measurement Information System
REDCap	Research Electronic Data Capture
STAT5	signal transducer and activator of transcription 5
Teff	effector T cell
TGF-β	transforming growth factor beta
TGF-β1	transforming growth factor beta 1
Treg	regulatory T cell
