# Supplementary material for: Low-Dose Interleukin-2 Enhances Activated Suppressor Regulatory T Cells and CTLA-4 and HLA-DR Expression in Chronic Chikungunya Arthritis
Source: Pathogens. 2026 Jul 22;15(7):770. doi: 10.3390/pathogens15070770 (PMC13416368; doi:10.3390/pathogens15070770)
Supplement: Supplementary file 1 [file pathogens-15-00770-s001.zip › pathogens-4414584-supplementary.pdf]

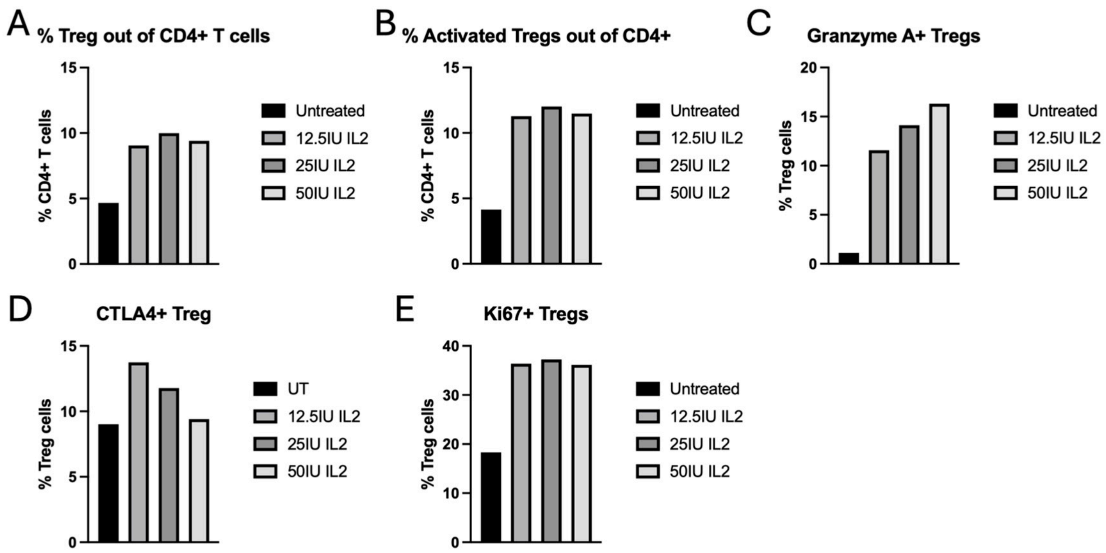

Figure S1: Determination of IL-2 concentration using healthy donor cells. Healthy donor PBMCs were treated with 12.5, 25, or 50 IU/ml IL-2, refreshed after 48 hours, and collected and stained for flow cytometry after 96 hours. Tregs (A), activated Tregs (B), Granzyme A+ Tregs (C), and Ki67+ Tregs (E) all increased with treatment compared to no treatment, with slightly greater changes in Tregs, activated Tregs, and Ki67 using 25 IU/ml. CTLA4+ Tregs (D) decreased with increasing amounts of IL2. Based on this data, 25 IU/ml was chosen for all subsequent experiments.

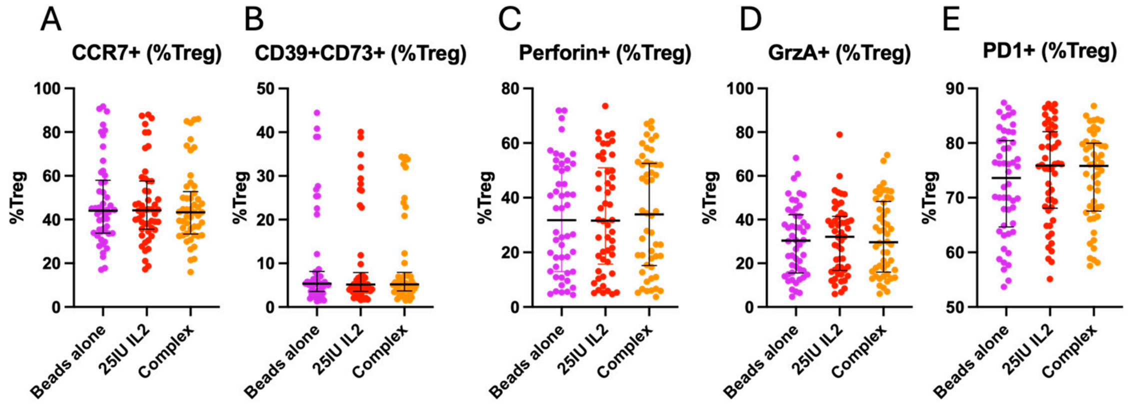

Figure S2: No effect of treatment on certain markers of Treg cell activity. Flow cytometry was performed on IL-2 or complex-treated CD4+ cells to analyze the effect of treatment on Treg cell activity, including CCR7 (A), CD38/CD73 (B), perforin (C), granzyme A (D), and PD-1 (E). Control wells were incubated with Treg Suppression Inspector beads alone. Lines on the graphs denote the median with IQR. Comparisons between groups were all non-significant.

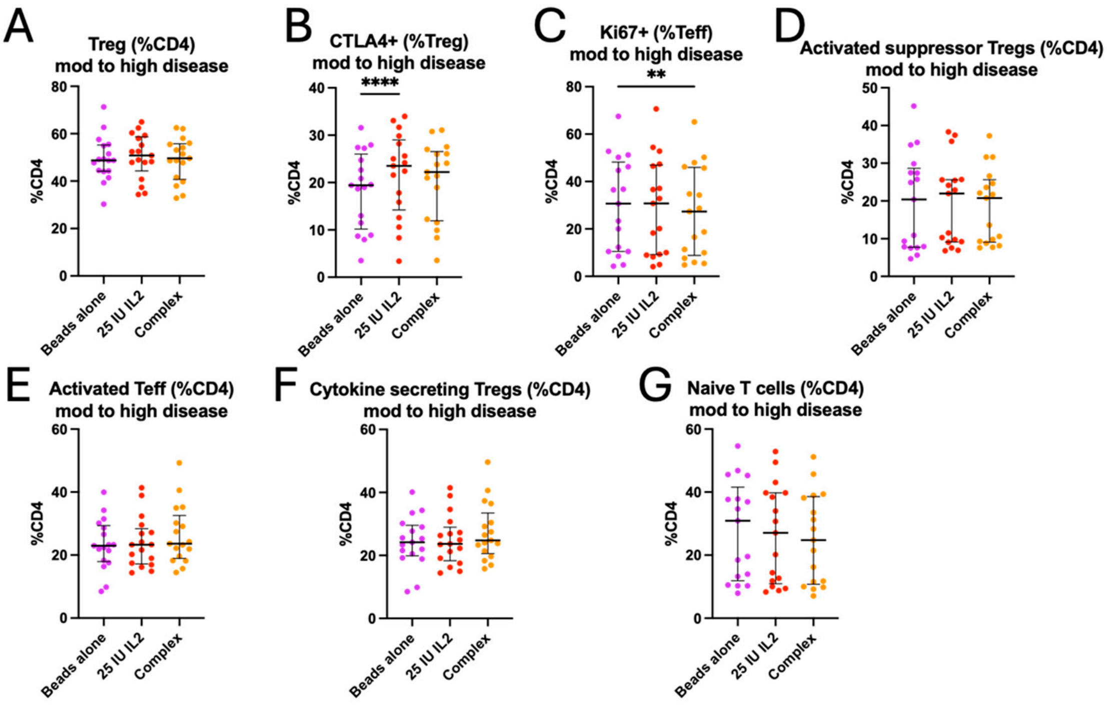

Figure S3: Effect of treatments on PBMCs from patients with moderate-to-high disease score. When observing only patients with moderate-to-high disease scores (DAS28 > 3.2), comparisons show an increase in CTLA4+ Tregs (B) when PBMCs were treated with IL2 and a decrease in Ki67+ Teff (C) with complex treatment compared to Treg Suppression Inspector beads alone. Other important populations that were analyzed, including total Tregs (A), activated suppressor Tregs (D), activated Teffs (E), cytokine-secreting Tregs (F), and naïve T cells (G), showed no significant changes from the beads alone control. Lines on the graphs denote the median with IQR.
